# Secretion modification region-derived peptide blocks exosome release and mediates cell cycle arrest in breast cancer cells

**DOI:** 10.18632/oncotarget.14513

**Published:** 2017-01-05

**Authors:** Ming-Bo Huang, Ruben R. Gonzalez, James Lillard, Vincent C. Bond

**Affiliations:** ^1^ Department of Microbiology, Biochemistry, and Immunology, Morehouse School of Medicine, Atlanta, Georgia, 30310, USA

**Keywords:** SMR peptide, PEG-SMR-Clu peptide, breast cancer, apoptosis, annexin

## Abstract

**Purpose:**

Discovery and development of a novel anticancer PEG-SMR-Clu peptide to prevent breast cancer metastasis. How breast cancer cells and primary mammary epithelial cells interact and communicate with each other to promote tumorigenesis and how to prevent tumor metastasis has long been a concern of researchers. Cancer cells secrete exosomes containing proteins and RNA. These factors can influence tumor development by directly targeting cancer cells and tumor stroma. In this study, we determined the effects of a peptide as an inhibitor of exosome secretion on breast tumors. We developed a peptide derived from the Secretion Modification Region (SMR) of HIV-1 Nef protein that was modified with PEG on the N-terminus and with a Clusterin (Clu)-binding peptide on the C-terminus. Attachment of PEG to the SMR peptide, termed PEGylation, offers improved water solubility and stability as well as reduced clearance through the kidneys, leading to a longer circulation time. The 12-mer Clu-binding peptide plays multiple roles in tumor development and metastasis. The Clu peptide can be detected by antibody *in vivo*, thus it has the potential to be used to monitor tumor status and treatment efficacy in animal studies and eventually in cancer patients.

**Results:**

PEG-SMRwt-Clu and PEG-SMRwt peptides inhibited the growth of both of MCF-7 (estrogen responsive, ER+) and MDA-MD-231 (estrogen non-responsive, ER-) human breast cancer cells in a dose and time-dependent manner, without inducing cytotoxic effects. The SMRwt peptide, combined with paclitaxel, induced G2/M phase cell cycle arrest on MCF-7 and MDA-MB-231 cells but did not promote apoptosis. PEG-SMRwt-Clu peptide treatment blocked exosome release from both MCF-7 and MDA-MB-231 cells. This effect was blocked by knockdown of the chaperone protein mortalin by either antibody or siRNA.

**Materials and methods:**

MCF-7 and MDA-MB-231 breast tumor cells were treated with PEG-SMR-Clu peptide alone and in combination with paclitaxel and cisplatin. Cell proliferation and viabilty were determined via cell cycle analysis using Cellometer imaging cytometry, Annexin V and MTT assays. The effects of the PEG-SMR-Clu peptide on tumor exosome release were determined by testing isolated exosome fractions, for (i) expression of CD63 and Alix proteins by Western blotting, (ii) NanoSight nanoparticle tracking analysis (NTA 10) to measure exosomes size and concentration, and (iii) measurement of acetylcholinesterase (AchE) for exosome specific enzyme activity.

**Conclusions:**

PEG-SMRwt-CLU peptides inhibited the growth of human breast cancer cells and blocked tumor exosome release *in vitro*. The peptide alone did not cause increased cytotoxicity or apoptosis induction, but did cause cell cycle G2/M phase arrest in both estrogen responsive and non-responsive breast cancer cells. These data suggest a potential therapeutic value of SMR to prevent breast cancer metastasis and as an adjuvant for the chemotherapeutic treatment of human breast cancer.

## INTRODUCTION

The role of exosomes in cancer development has become the focus of much research. Exosomes are microvesicles released from many cell types, but are secreted in substantially higher concentrations from cancer cells [1–3]. In recent years, exosomes have been found to play roles in many cellular functions, particularly in cancer. Exosomes from cancer cells can affect tumor microenvironment, tumorigenesis and metastasis [4–9].

According to the American Cancer Society (ACS), an average of 232,340 cases is diagnosed with 39,620 deaths from breast cancer each year. An estimated 2,350 men are diagnosed with breast cancer with estimated 440 expected to die. Furthermore, a cure has not been developed for metastatic breast cancer (www.cancer.org).

Treatments of advanced breast cancer include surgery, radiotherapy and/or chemotherapy, with the latter being most commonly used, but the clinical efficacy of chemotherapy has been limited by serious side-effects, toxicity and drug resistance [10]. Therefore, new methods for preventing the spread of malignant tumors are focused on treatment with high efficacy and low toxicity, with a change from the traditional chemical approach to a more molecular approach of pharmacotherapy. The key point is that traditional chemicals not only act on malignant tumor cells but also attack normal cells, causing significant damage to DNA, RNA, microtubules and other components which are of vital importance to the cells. Thus, the older drugs are now gradually being replaced by new, bio-targeted anti- tumor drugs [11]. Our group has investigated a series of Nef peptides as agents to induce tumor cells apoptosis [12–16, 52] We have developed peptides from the secretion modification region (SMR) of HIV-1 Nef as a promising source of effective, less toxic new anticancer drugs.

We propose that SMR peptides could be new biological agents for the prevention and treatment of breast cancer. Breast tumor cells have been found to secrete, in a regulated manner, exosomes that carry tumor antigens. Additionally, secretion of exosomes by tumor cells can help to present antigens or transmit them to antigen presenting cells [17, 18]. These tumor-released exosomes cause immune suppression through immune cell killing or dysregulation, thereby promoting a state of immunosuppression that allows for rapid tumor growth [19, 20]. We have shown that HIV-1 viral protein Nef is involved in the exosomal pathway of normal cells that promotes a state of immune privilege/suppression which ultimately could lead to Human Immunodeficiency Virus (HIV) and Acquired Immune Deficiency Syndrome (AIDS). Moreover, we have previously shown that HIV-1 Nef protein expression induces changes in trafficking, and leads to secretion of Nef-containing exosome-like vesicles which are capable of dysregulating or even killing immune cells [21–23]. We have analyzed the genetics of Nef-induced secretion, identifying specific motifs on Nef protein that interact with components of the endosomal trafficking machinery, allowing Nef to be sorted into Multivesicular Bodies (MVB) and packaged into exosomes [24]. The HIV-1 Nef SMR motif binds to the cellular chaperone protein mortalin. Disruption of this interaction inhibited both the secretion of exosomal Nef (exNef) and the release of the virus [25, 53]. This work led to development of a small peptide antagonist containing a Nef secretion domain (PEG-SMRwt-Clu), which, when taken into cells, can block Nef-induced exosome secretion from those cells.

In this study, we tested whether the PEG-SMRwt-CLU peptide could effectively inhibit the growth of human breast cancer cell lines and whether this effect is accompanied by blocking secretion of exosomes from the tumor cells. In addition, we tested whether the SMR peptide might have adjuvant effects for chemotherapeutic drugs. The data presented suggest that the SMR peptide inhibited breast cancer cell growth, reduced exosome secretion without increasing the cytotoxic effects of chemotherapy or promoting apoptosis.

## RESULTS

### SMR peptides inhibited cell growth of breast cancer cells

**Figure 1:**
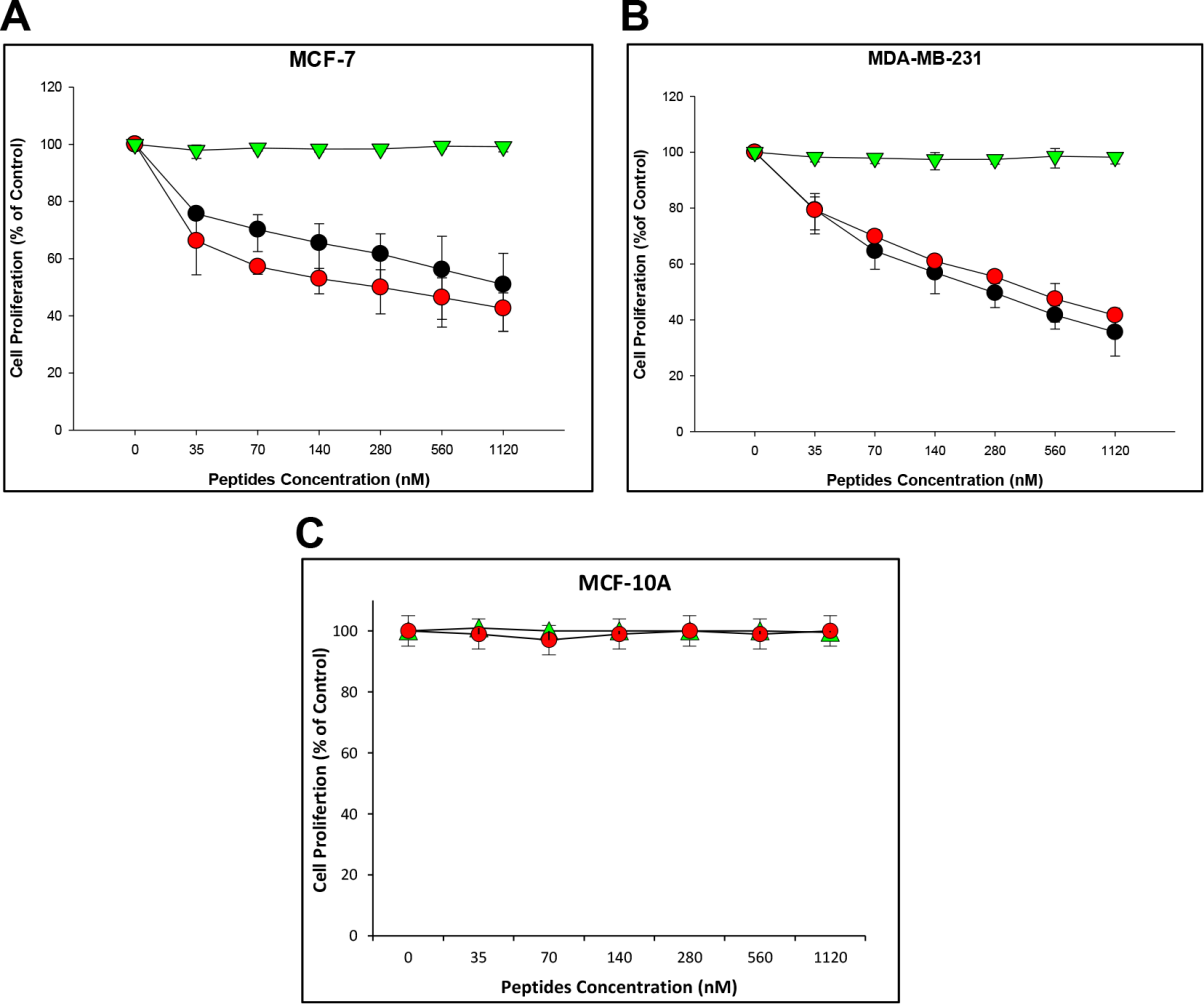
SMRwt peptide antagonists inhibit proliferation of MCF-7 and MDA-MB-231 breast cancer cells but not non-tumorigenic cells Cells were incubated with peptides at varying dosage (0–1120 nM) for 24 hr, after which proliferation was measured by MTT assay. Results of three independent experiments are shown. (**A**) Proliferation of MCF-7 breast cancer cells. (**B**) Proliferation of MDA-MB-231 breast cancer cells. (**C**) Proliferation of non-tumorigenic MCF-10A cells. Red dots indicate PEG-SMRwt peptide, black dots indicate PEG-SMRwt-CLU peptide, and green triangles indicate PEG-SMRmut peptide.

Breast cancer cells were treated for 24 hours with increasing concentrations (35 nM/mL, 70 nM/mL, 140 nM/mL, 280 nM/mL, 560 nM/mL and 1120 nM/mL) of the PEG-SMRwt-Clu peptide and either PEG-SMRwt, or PEG-SMRmut peptides as controls, both peptides containing the SMRwt sequence inhibited breast cancer cell growth in a dose-dependent manner (Figure 1). For MCF-7 cells, 50% inhibition was seen at 1.12 μM/mL of PEG-SMRwt-Clu, and 0.28 μM/mL of PEG-SMRwt. For MDA-MB-231 cells 50% inhibition was achieved with 0.28 μM/mL of PEG-SMRwt-Clu and 0.42 μM/mL of PEG-SMRwt. The PEG-SMRmut peptide did not inhibit proliferation.

### SMRwt peptides contribute to cell cycle arrest in breast cancer cells

**Figure 2:**
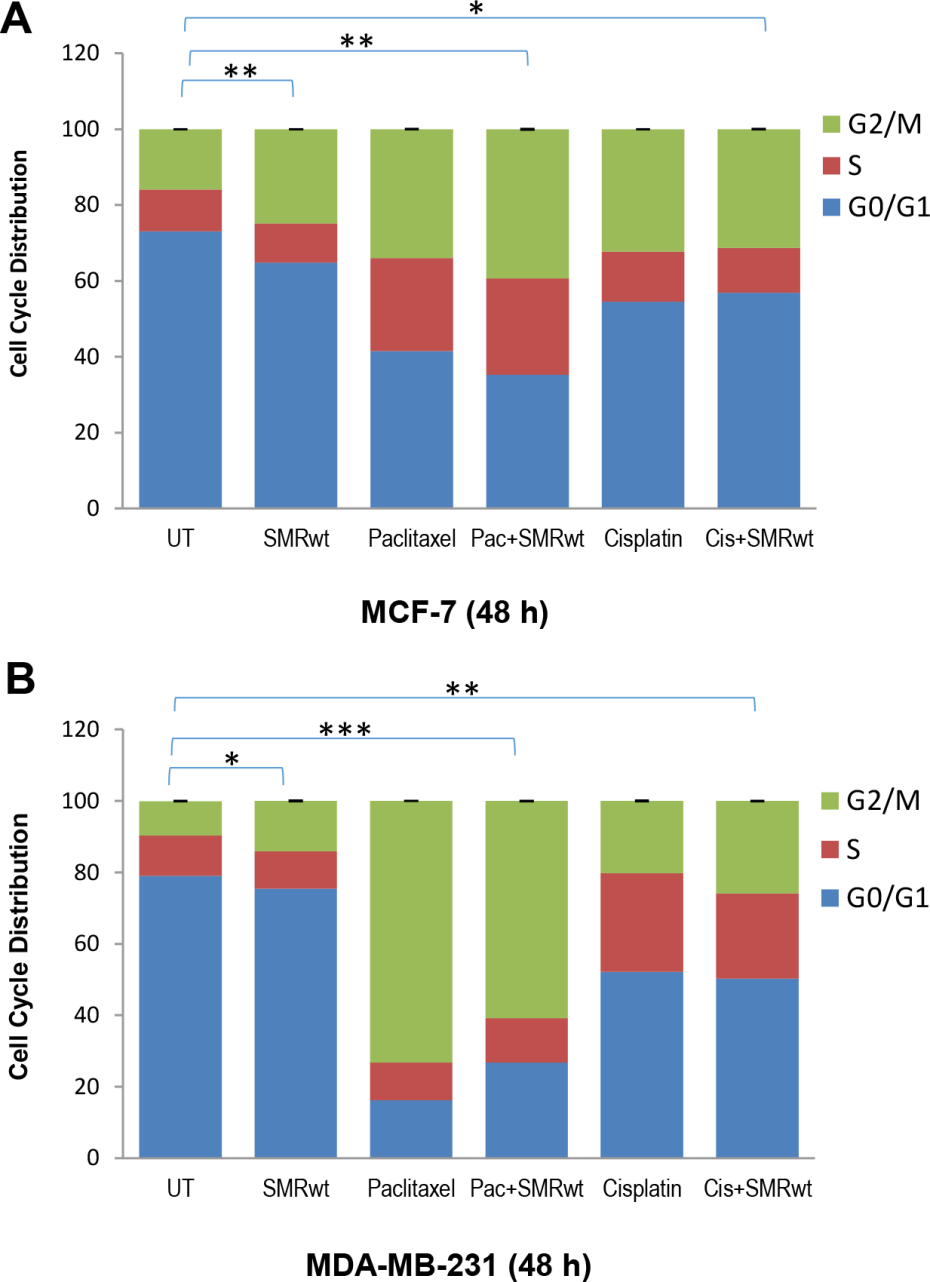
PEG-SMRwt-CLU peptide antagonist and chemotherapeutics induced cell cycle arrest in MCF-7 and MDA-MB-231 breast cancer cells Cells were treated 48 hr with SMR peptides, alone or in combination with paclitaxel or cisplatin, and were assayed by Cellometer imaging cytometry, indicating percentage of MCF-7 (**A**) and MDA-MB-231 (**B**) in various cell cycle phases. Results of two independent experiments are shown. Significant differences relative to untreated control are indicated as follows: *p < 0.01, **p < 0.001 for MCF-7 cells, and *p < 0.02, **p < 0.01, ***p < 0.0001 for MDA-MB-231 cells.

The data indicated that PEG-SMRwt-CLU peptides induced cell cycle arrest in MCF-7 cells and MDA-MB-231 cells assayed at 48 hours (Figure 2). When cells were treated with the PEG-SMRwt-CLU peptide, or the peptide combined with paclitaxel or cisplatin, they were blocked in G2/M phase, indicating that PEG-SMRwt-Clu peptides contribute to induction of G2/M arrest in breast cancer cells.

### SMRwt peptides increased the sensitivity of breast cancer cells to cisplatin and paclitaxel in MCF-7 breast cancer cells

**Figure 3:**
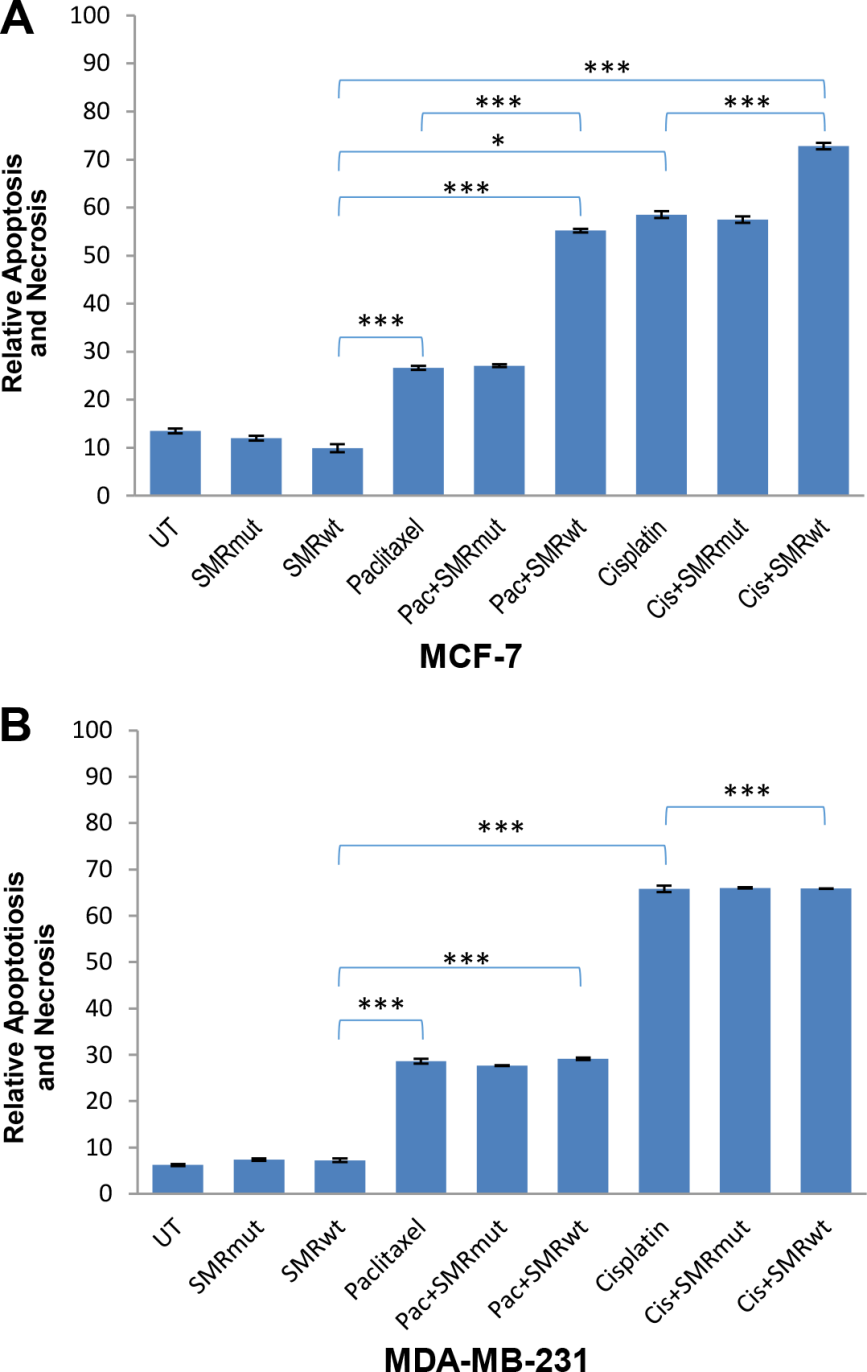
PEG-SMRwt-CLU peptide antagonist increased cytotoxicity in MCF-7 but not in MDA-MB-231 breast cancer cells Percentage of apoptotic cells (**A**) MCF-7 and (**B**) MDA-MB-231, as determined by Annexin V-FITC assay of cells treated for 48 hr with peptide alone or combined with paclitaxel or cisplatin. Error bars represent mean ± SD of four independent experiments. Significant differences relative to SMRwt peptide are indicated as follows: *p < 0.01, ***p < 0.0001.

In a separate experiment, MCF-7 and MDA-MB-231 cells were treated with PEG-SMRwt-CLU and PEG-SMRmut-CLU, alone and in combination with paclitaxel or cisplatin and assayed for apoptosis by Annexin V-FITC/PI assay. Both of the cell lines showed increased apoptosis relative to the SMR peptides alone after the incubation with paclitaxel and cisplatin for 48 hours (Figure 3). Interestingly, while the PEG-SMRwt-Clu peptide was not apoptotic by itself, it increased the level of drug-induced apoptosis in MCF-7 cells, which was not seen in MDA-MB-231 cells. An important difference in these cell lines is that MCF-7 cells express the estrogen receptor (ER), while MDA-MB-231 cells are ER-negative. The increased cytotoxicity of peptide in the presence of the drugs merits further investigation.

### SMR peptide blocked exosomes release in breast cancer cells

We performed acetylcholinesterase (AchE) assays, NanoSight analysis and Western blot analysis to characterize exosomes released from MCF-7 and MDA-MB-231 human breast cancer cells treated for 48 hr with the various peptides. The results indicated that exosome release was inhibited by the PEG-SMRwt-CLU peptide.

**Figure 4:**
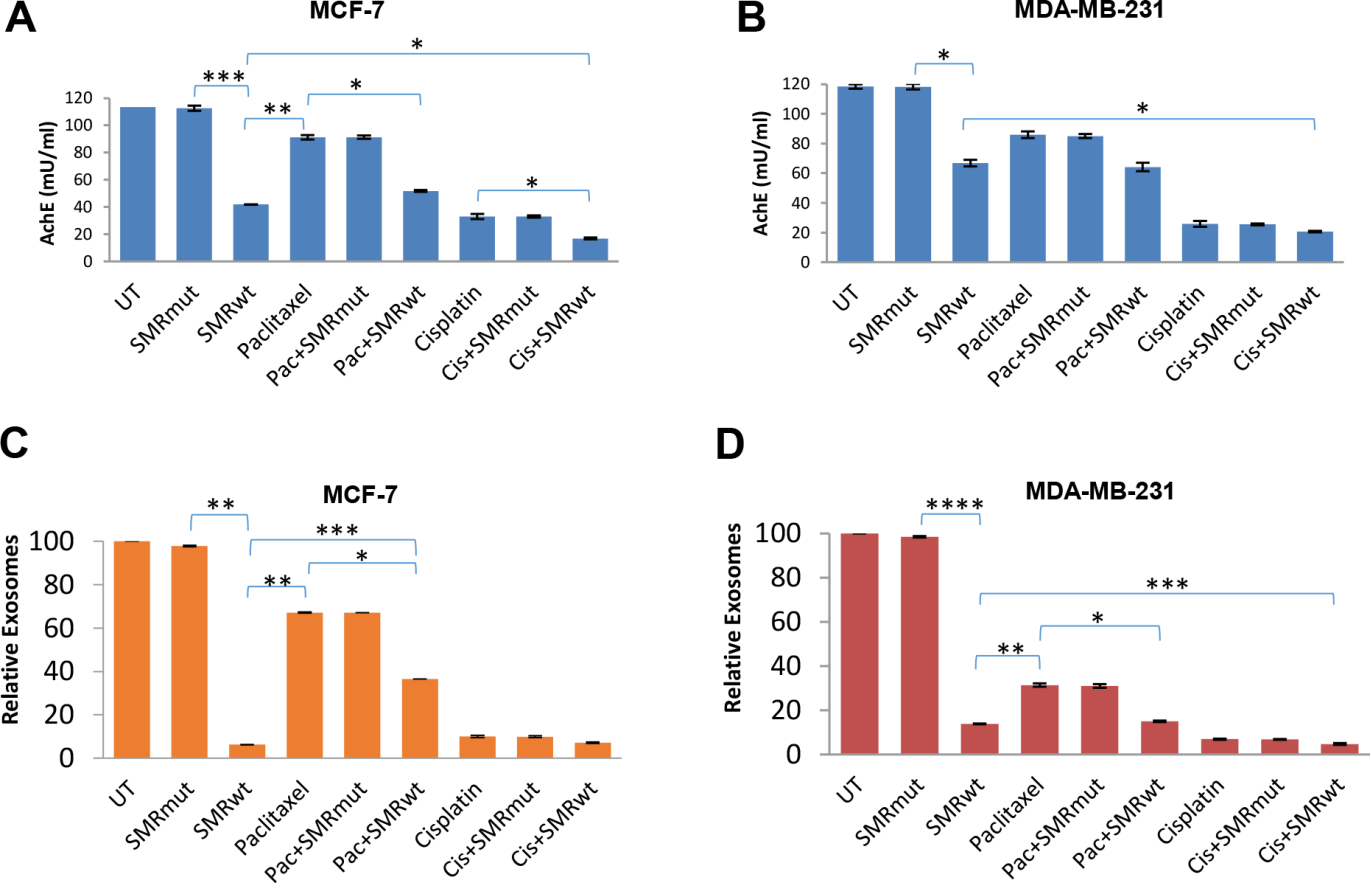
PEG-SMRwt-CLU peptide antagonist blocks exosome release from MCF-7 and MDA-MB-231cells Cells were treated for 48 hr with peptide alone or combined with paclitaxel or cisplatin. (**A, B**) Relative level of exosomes released from MCF-7 and MDA-MB-231 cells respectively, determined by AchE assay. Error bars represent mean ± SD of four independent experiments. Significant differences relative to SMRwt peptide: *p < 0.01, **p < 0.001, ***p < 0.0001 for MCF-7 cells; and *p < 0.01 for MDA-MB-231 cells. (**C, D**) Relative numbers of exosomes released by MCF-7 and MDA-MB-231 cells respectively, as determined by Nanosight measurement. Error bars represent mean ± SD of two independent experiments. Significant differences relative to SMRwt peptide: *p < 0.01, **p < 0.001, ***p < 0.0001 for MCF-7 cells and *p < 0.03, **p < 0.02, ***p < 0.01 and ****p < 0.001 on MDA-MB-231 cells.

First, AchE activity in exosomes was assayed. For MCF-7 cells, (Figure 4A) the control exosomes contained 113.49 mU/mL of AchE activity compared to 41.95 mU/mL of activity for the PEG-SMRwt-CLU peptide alone, 51.87 mU/mL for paclitaxel combined with PEG-SMRwt-CLU, and 16.95 mU/mL for cisplatin combined with PEG-SMRwt-CLU (Figure 4A). For MDA-MB-231 cells (Figure 4B), control exosomes contained 118.48 mU/mL of AchE activity compared with 66.77 mU/mL for the PEG-SMRwt-CLU peptide alone, 64.15 mU/mL activity for paclitaxel combined with PEG-SMRwt-CLU and 27.0 mU/mL activity for cisplatin combined with PEG-SMRwt-CLU.

Second, analysis of concentration and size distribution of exosomes was assayed using NanoSight LM10 Nanoparticle Tracking Analysis (NTA). With NTA, particles are automatically tracked and sized, based on Brownian motion and the associated diffusion coefficient. Before analysis of the samples by NTA, we made sure that salt aggregates from PBS did not contribute to background and that equipment was free of contaminant particles. For MCF-7, untreated controls contained 5.16 × 10^9^ particles/mL (Figure 4C). For MDA-MB-231 the control cultures contained 4.7 × 10^9^ particles/mL (Figure 4D). However, the treatment with PEG-SMRwt-CLU (3.28 × 10^8^ particles/mL, *p* < 2.40E-06), PEG-SMRwt-CLU/paclitaxel (5.7 × 10^8^ particles/mL, *p* < 0.0008) and PEG-SMRwt-CLU/cisplatin (3.77 × 10^8^ particles/mL, *p* < 0.0001) were lower than the control (Figure 4C). For MDA-MB-231, concentration of exosomes from PEG-SMRwt-CLU alone was 6.8 × 10^8^ particles/ml, (*p* < 3.96E-05), while PEG-SMRwt-CLU/paclitaxel (7.5 × 10^8^ particles/mL, *p* < 0.001), and PEG-SMRwt-CLU/cisplatin (3.06 × 10^8^ particles/mL, *p* < 5.37E-05) were lower than control (Figure 4D). For all cultures, NTA estimated the size of the exosomes to be in the range of 30 to 47 nm.

**Figure 5:**
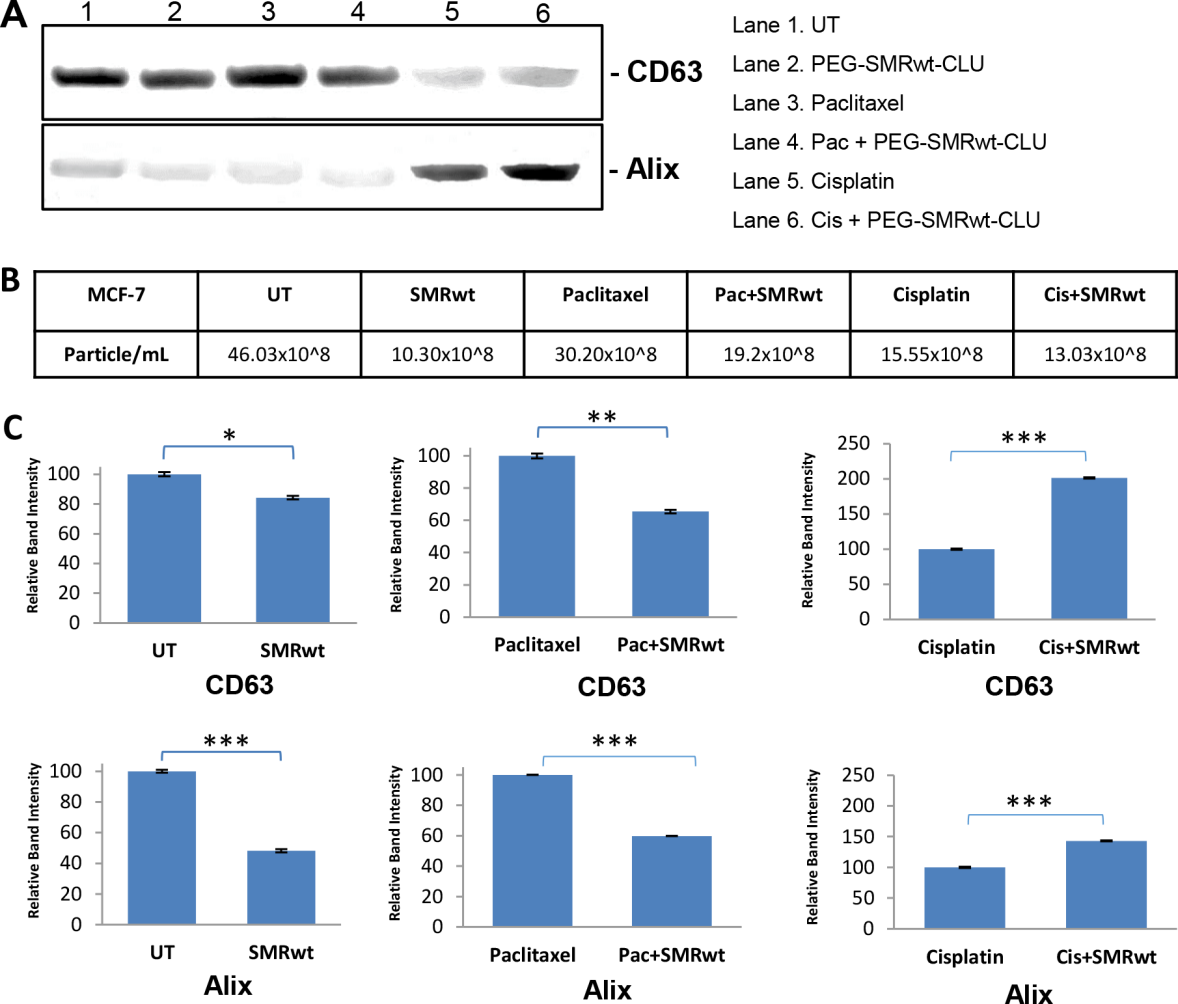
Exosome-specific proteins can be detected on exosomes from MCF-7 breast cancer cells Cells were treated for 48 hr with SMRwt peptide alone or combined with paclitaxel or cisplatin. (**A**) Expression of exosome proteins by Western blot analysis and (**B**) Exosome numbers were measured by NanoSight and (**C**) Densitometry analysis showing relative intensity of bands. Data represent the mean ± SD of three independent experiments. Significant differences relative to treatment with peptide are indicated as follows: *p < 0.01, **p < 0.001, ***p < 0.0001.

**Figure 6:**
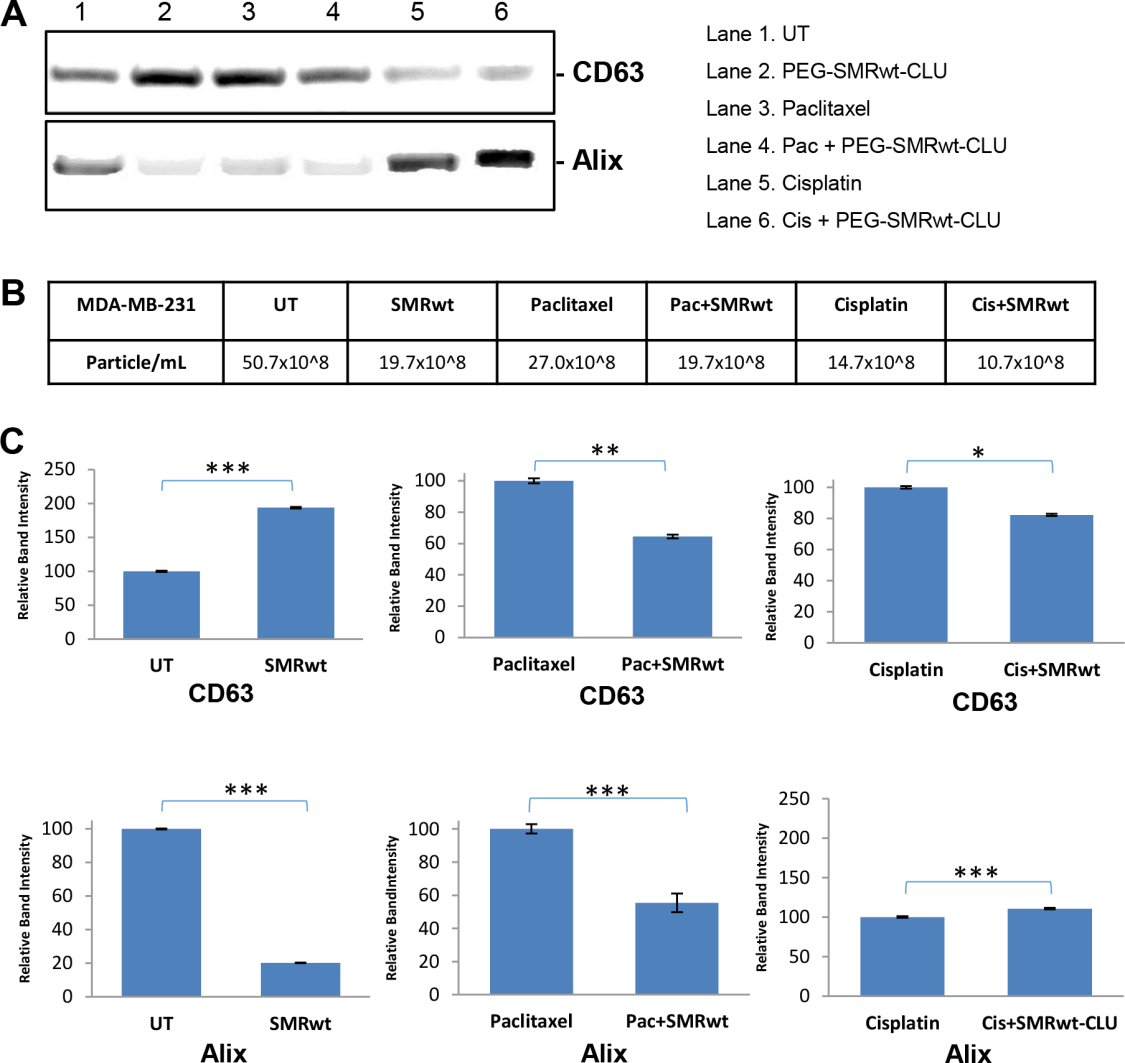
Exosome-specific proteins can be detected on exosomes from MDA-MB-231 breast cancer cells Cells were treated for 48 hr with SMRwt peptide alone or combined with paclitaxel or cisplatin. (**A**) Expression of exosome proteins by Western blot analysis and (**B**) Exosome numbers were measured by NanoSight and (**C**) Densitometry analysis showing relative intensity of bands. Data represent the mean ± SD of three independent experiments. Significant differences relative to treatment with peptide are indicated as follows: *p < 0.01, **p < 0.001, ****p < 0.0001.

Finally, we used Western blot analysis to detect exosome proteins in controls and peptide-treated cultures. Western blot analysis revealed the presence of human CD63 and Alix markers in the all exosomes isolated from MCF-7 cells (Figure 5) and MDA-MB-231 cells (Figure 6). For both cell types the numbers of exosomes were decreased in all the cultures treated with the PEG-SMRwt-CLU peptide. The relative amounts of Alix and CD63 exosome markers were variable, however. The CD63 level in MCF-7 cells was increased when PEG-SMRwt-CLU was added with cisplatin compared to cisplatin alone (Figure 5). CD63 was also increased in MDA-MB-231 cells with the peptide as compared to untreated cells. This suggests that exosome numbers and exosome composition are regulated differently.

### Blocking the SMR-mortalin interaction blocks exosome release in breast cancer cells

**Figure 7:**
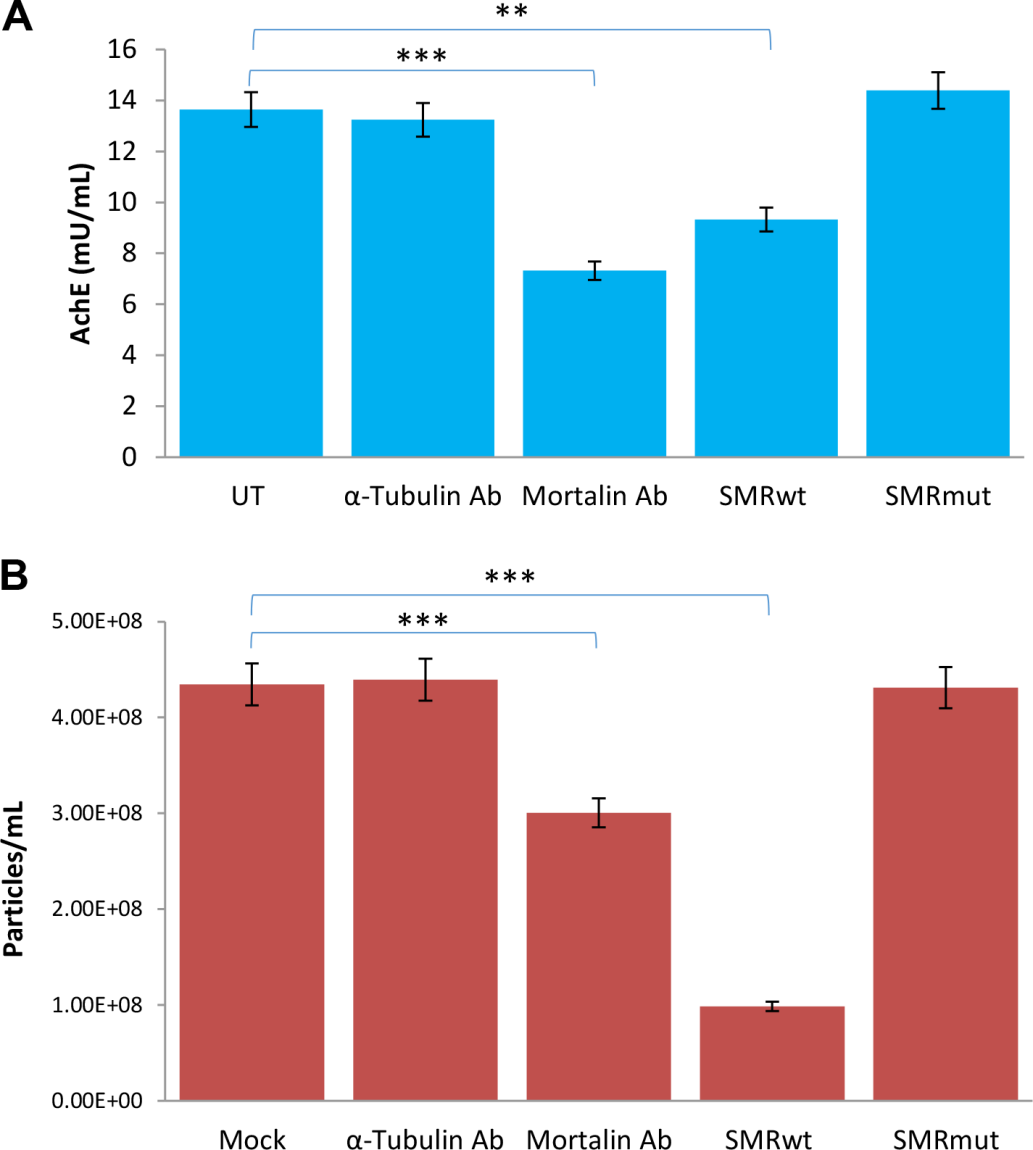
Antibody to mortalin inhibits exosome secretion from MCF-7 breast cancer cells MCF-7 cells were either transfected with antibodies to mortalin or alpha-tubulin, or treated with SMRwt or SMRmut peptides. (**A**) Relative exosome release level after 48 hr by AchE assay. (**B**) Relative numbers of exosomes released after 48 hr by NanoSight analysis. Error bars represent the mean ± SD of three independent experiments. Significant differences relative to untreated cells: *p < 0.0001, **p < 0.0001.

We previously identified the HSP70 family protein mortalin as a binding partner for the HIV-1 Nef SMR, and showed that disruption of SMR-mortalin binding interfered with exosome release in lymphocytes [25]. To test whether this can be a mechanism for the observed SMR peptide effect on exosome release from breast cancer cells, we transfected MCF-7 cells with either antibody to mortalin or antibody to α-tubulin (as a control). The anti-mortalin treated cells were significantly impaired in exosome release as measured by AchE assay (Figure 7A) and slightly less affected when measured by NTA assay (Figure 7B). The effect of anti-mortalin was similar to that observed for treatment of MCF-7 cells with the PEG-SMRwt-CLU peptide.

**Figure 8:**
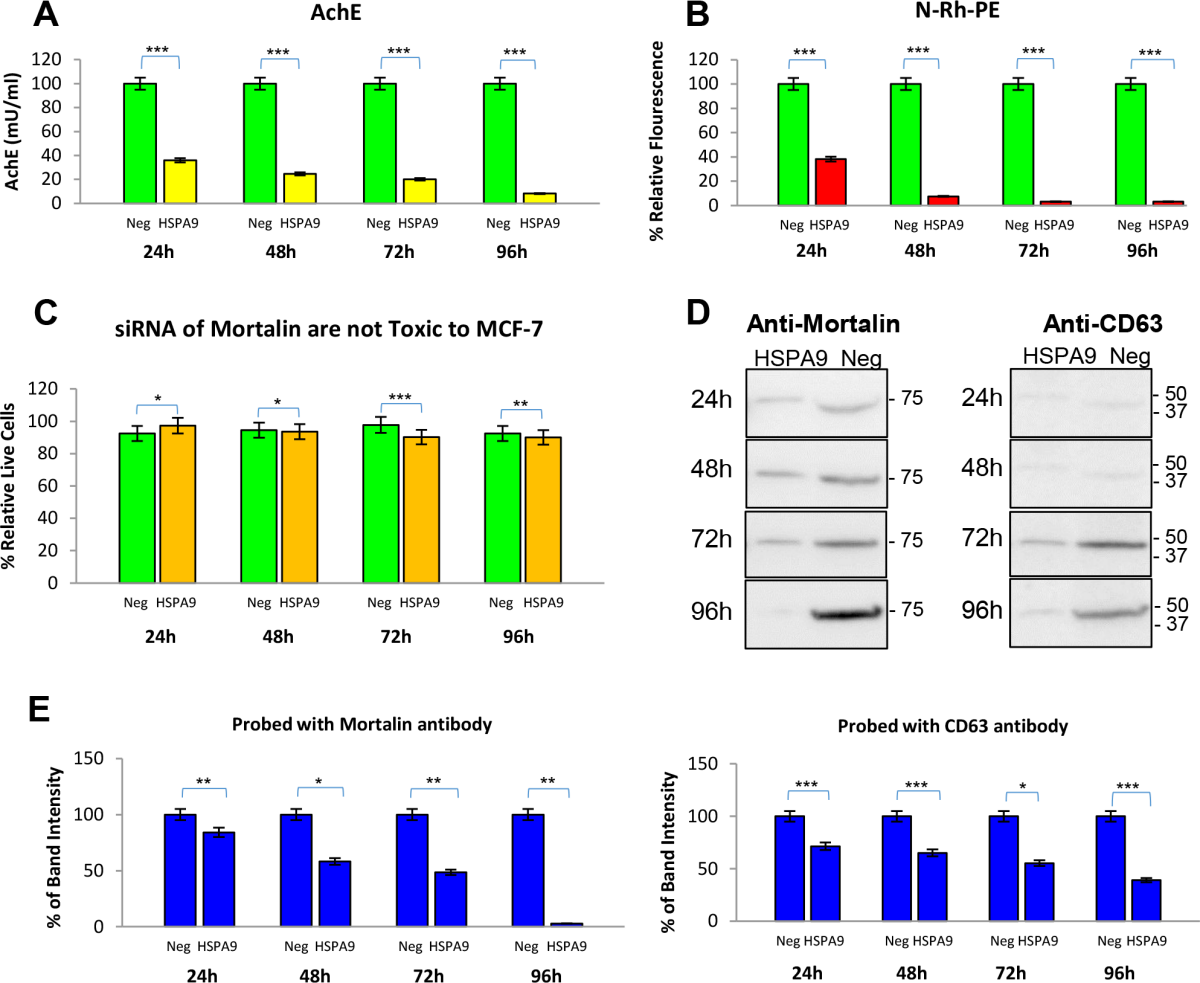
Exosome secretion is decreased in MCF-7 breast cancer cells by knockdown of mortalin expression MCF-7 cells were transfected with clones expressing siRNA against either mortalin (HSPA9) or a negative control RNA. Exosomes were isolated and analyzed after 24, 48, 72, and 96 hr for changes in (**A**) level of exosome secretion by AchE assay. Significant differences relative to controls are indicated: ***p < 0.0001 (**B**) exosome secretion by N-Rh-PE. Significant differences relative to controls are indicated: ***p < 0.0001, (**C**) percentage of live cells remaining at each time point, *p < 0.05, **p < 0.002, ***p < 0.0001 (**D**) mortalin and CD63 protein expression levels by Western blotting. (**E**) Densitometry analysis of Western blot data. Significant differences relative to controls are indicated: *p < 0.01, **p < 0.001, ***p < 0.0001.

Next, we knocked down expression of the mortalin protein by transfecting MCF-7 cells with a clone designed to express a siRNA against mortalin (HSPA9). The siRNA blocked exosome secretion, by AchE and membrane fluorescence (N-Rh-PE) assays, at all-time points tested (Figure 8A, 8B), without observed cell toxicity (Figure 8C). The exosomes from the siRNA transfected cells were assayed by Western blotting for mortalin and for the exosome marker CD63, a tetraspanin. Expression of both mortalin and CD63 was significantly decreased at 48 hr, and the decline in expression for both proteins continued through 96 hr (Figure 8D, 8E).

## DISCUSSION

The Secretion Modification Region (SMR; ^66^VGFPV^70^) of HIV-1 Nef is critical for Nef's induction of exosome secretion. SMR peptides displayed very reduced or abolished Nef-induced secretion after alanine replacement of any of its five amino acids [22, 25]. Computer modeling studies done by our group indicated the SMR forms a putative binding pocket that is severely disrupted by single amino acid changes to the primary sequence [24]. Subsequent studies identified the cellular protein mortalin as a Nef binding partner, interacting with the SMR [25]. Further, disruption of this interaction by a SMR-derived peptide (SMRwt) inhibits both the secretion of exNef and the release of virus [25]. In the current study, we have further developed the SMR peptide by modification with PEG and a clusterin-binding peptide (Clu), and characterized the PEG-SMRwt-Clu peptide's effect on breast cancer cells.

Attachment of PEG to the SMR peptide, or PEGylation, offers improved water solubility and stability as well as reduced clearance through the kidneys, leading to a longer circulation time. We also added a Clusterin-binding sequence. Clusterin, a large glycoprotein of the heat-shock family, is known to stabilize secreted proteins, and has been dectected in many different cell types and in extracellular fluids [26]. The 12-mer Clusterin binding peptide can be easily detected by antibody-based assays, thus offering the opportunity to monitor both uptake and metabolism of Clu-modified peptides.

A potential mechanism of PEG-SMRwt-Clu effects on breast cancer cells may involve interaction with mortalin or with cyclins. The peptide is thus being proposed as an effective agent for the prevention or treatment of human breast cancers, as identified in the cell cycle, dose response and MTT assays. Additionally, we have shown a role for the peptide in exosome secretion by AchE assay, NanoSight analysis and Western blotting assays. In the present study, we found PEG-SMRwt-CLU could inhibit the growth of both of MCF-7 and MDA-MB-231 of human breast cancer cells *in vitro*. However, treatment with SMR peptide alone only inhibited cell growth of MCF-7 and MDA-MB-231 breast cancer cells, but did not induced the apoptosis of either type of breast cancer cells. This effect of the PEG-SMRwt-CLU peptide on exosome release in breast tumor cells is related to its interaction with mortalin, and this could be related to p53, STAT3 or cyclin functions related to tumor exosome release. Interestingly, a low dose (0.28 μM) of PEG-SMRwt-CLU peptide significantly enhanced the effects of cisplatin on breast cancer cells MDA-MB-231. These data suggest the further potential value of SMR peptides targeting mortalin for the prevention or treatment of human breast cancer.

Overexpression of HSP70s is thus associated with tumor transformation and eventually results in a decrease of chemotherapy efficacy. Notably, the distribution of HSP70s is deregulated in cancer cells. It has been reported that HSP70s localize distinct organelles or are exported to humoral circulation during cancer development. Either surface or exported HSP70s play danger signals and trigger immune response to destroy the tumor cells. It has been found that the HSP70s prevent stress induced apoptosis through either mitochondria dependent or independent pathways. HSP70s can block activation of the death factors to allow the cells resistance to stress-induced apoptosis. Overexpression of mortalin in a wide variety of malignancies is generally associated with highly advanced and aggressive cancers [27]. The results of previous and current studies suggest that mortalin may be an HSP70s family protein interacting with our modified SMR peptide.

A potential mechanism involved in PEG-SMRwt-Clu actions on breast cancer cells includes its interaction with the chaperone protein mortalin. Mortalin is overexpressed in several cancer cell lines, e.g. human brain tumors, hepatitis C virus-related hepatocellular carcinoma, SH-SY5Y neuroblastoma, A549 lung adenocarcinoma, LoVo colon adenocarcinoma, and Sup-B15 acute lymphoblastic leukemia (B cell) cell lines and ovarian tumor cells etc. [28–31]. The direct link between mortalin and these disease states remains uncertain; however, it has been identified as a potential therapeutic target or biomarker. Mortalin resides in multiple cellular locations that include the endoplasmic reticulum, cytoplasmic vesicles, and the cytosol, but primarily is found in mitochondria. Moreover, mortalin has been associated with multiple functions, including intracellular trafficking, antigen processing, regulation of cell proliferation, aging, differentiation and tumorigenesis [32, 33], while also interacting with many other partner proteins, including the adaptor protein Tim44 in the mitochondrial protein import machinery [34, 35]. (*ii*) PEG-SMRwt-CLU peptide interaction Cyclin Dependent Kinase 1–cyclin B (CDK1-cyclin B), CDK1-cyclin B is a member of cyclin-dependent kinases implicated in cell cycle control in eukaryotes. Activation of the CDK1-cyclin B brings the onset of mitosis and is tightly regulated. During G2 phase, CDK1-cyclin B complex is held in an inactive state by phosphorylation of CDK1 at the two negative regulatory sites, T14 and Y15 by CDK1 inhibitory protein kinases, Myt1 and Wee1 respectively. Dephosphorylation of T14 and Y15 by cell division cycle (CDC25) protein phosphatase in late G2 phase activates the CDK1-cyclin B complex and triggers the initiation of mitosis. During expression in insect cells, the recombinant CDK1-cyclin B is activated *in vivo* by endogenous kinase [36–38]. We hypothesize PEG-SMRwt-CLU peptide might be involved CDK1-cyclin B functions, and induced G2/M phase arrest. They will be identifying in further, and (*iii*) PEG-SMRwt-CLU peptide interaction p53. The p53 tumor suppressor protein plays a major role in cellular response to DNA damage and other genomic aberrations. Activation of p53 can lead to either cell cycle arrest and DNA repair or apoptosis [39]. p53 is phosphorylated at multiple sites *in vivo* and by several different protein kinases *in vitro* [40, 41]. Phosphorylation of p53 at Ser392 is increased in human tumors [42] and has been reported to influence the growth suppressor function, DNA binding, and transcriptional activation of p53 [43–45]. Phosphorylation of p53 at Ser46 regulates the ability of p53 to induce apoptosis [46, 53]. PEG-SMRwt-CLU peptide might be bind to mortalin and blocking p53 to induce apoptosis.

In the future studies, it will be important to investigate the molecular mechanism of the PEG-SMRwt-CLU peptide and exosomes in cancer both *in vitro* and via a mouse model *in vivo* to confirm its efficacy.

In summary, we have shown that a HIV-Nef SMR-derived peptide inhibited the growth of human breast cancer cells by inducing tumor cell cycle arrest and blocking tumor exosomes release. The SMR peptide inhibits the tumor cell cycle at G2/M phase boundary. When the SMR peptide and chemotherapeutic drugs were combined to treat tumor cells, PEG-SMRwt-CLU synergistically potentiated the anti-proliferative effects of the drugs, significantly enhanced the growth inhibitory effect of drugs and blocked exosomes release both MCF-7 and MDA-MB-231 breast cancer cells, further indicating the potential clinical application of PEG-SMRwt-CLU peptide for the prevention and treatment of human breast cancer.

## MATERIALS AND METHODS

### Cell lines, reagents and antibodies

The MCF-7 cell line, a noninvasive estrogen receptor positive (ER+) and MDA-MB-231 cell line ER negative were purchased from the American Type Culture Collection (ATCC, Manassas, VA). MCF-10A cell line, a non-tumorigenic epithelial cell line was also purchased from ATCC. 3-(4,5-dimethylthiazol-2-yl)-2, 5-diphenyltetrazolium (MTT), Dulbecco's Modified Eagle's Medium (DMEM) with high glucose and FluoroBrite^™^ phenol red-free DMEM, (MCF-7) were purchased from Thermo Fisher Scientific (Rockford, IL) The RPMI 1640 medium (MDA-MB-231 cells) was obtained from Life Technologies Company (Carlsbad, CA). The basal medium MEBM and the additive MEGM (MCF-10A cells) were obtained from Lonzal/Clonetics Corporation (Lonza, Walkersville, MD). Paclitaxel was purchased from Sellck-Chemon, (Houston, TX). Cisplatin was purchased from EMD/Millipore (Billerica, MA). Annexin V-FITC/PI Apopto and PI Cell Cycle Kits were purchased from Nexcelom Bioscience (Lawrence, MA). The CD63 Rabbit polyclonal and Alix goat polyclonal antibodies were purchased from Santa Cruz Biotechnology Inc. (Santa Cruz, CA). The SMRwt and SMRmut peptide sequences are described in [25]. For these experiments, peptides were mofdified by addition of polyethylene glycol (PEG) and a Clusterin-binding peptide. The PEG-SMRwt-Clu PEG-SMRwt and PEG-SMRmut HIV-1 Nef peptides were synthesized by the InnoPep Company (San Diego, CA).

### Cell culture

Cells were cultured in the media described above with addition of exosome-free fetal bovine serum (System Biosciences Inc. Mountian View, CA), 100 units/mL penicillin, and 100 mg/mL streptomycin and maintained in a humidified atmosphere at 37^°^C and 5% CO2.

### Viability and cell proliferation assay

Human breast cancer cell lines MCF-7 and MDA-MB-231, and non-tumorigenic MCF-10A cells were seeded into 96-well plates (5000 cells/well) and treated for 24 hours with various concentrations of SMR peptides including PEG-SMRwt-Clu, PEG-SMRwt and PEG-SMRmut to determine IC50 (inhibition concentration). Cell proliferation was determined using the MTT dye assay (Molecular Devices. Sunnyvale, CA). Control experiments were performed with MTT treated cells alone and untreated cells, and on this basis, the incubation times of 24 hr and 48 hr, was used for MTT assays of peptide-treated cells [47, 48].

### Cell cycle analysis

MCF-7 and MDA-MB-231 cells were cultured separately by seeding in 6-well plates at 4 × 10^5^ cells per well. Cells were treated with either paclitaxel or cisplatin, either alone or combined with the PEG-SMRwt-CLU peptide for 48 hours. Cell cycle analysis was performed using a propidium iodide-based cell cycle assay and measured using Cellometer (Nexcelom, MA). For treatment of cells with peptides predetermined IC50 peptide concentrations were used: 1.12 μM of PEG-SMRwt-Clu, for MCF-7 cells for 24 hours and 0.28 μM of PEG-SMRwt-Clu, for MDA-MB-231 cells for 24 hours. Breast cancer cells were seeded into 96-well plates at 5 × 10^3^ cells/ml, and treated with either 1.6 μM/mL of paclitaxel, or 3 mg/mL cisplatin, 1.12 μM/mL PEG-SMRwt-CLU peptide, SMR peptide combined with paclitaxel or with cisplatin (MCF-7 cells). Alternatively 1.6 μM/mL paclitaxel or 2 mg/mL cisplatin or 0.28 μM/mL of PEG-SMR-CLU peptide, or the peptide combined with each of these drugs was used forMDA-MB-231 cells. The concentrations for cisplatin and paclitaxel were also the pre-determined IC50 dosages for the different cell types. These concentrations for the peptides and drugs were used in all subsequent experiments. After 48 hr incubation the cells were assessed by the Cellometry imaging cytometry assay. In order to test whether the peptide and chemical drugs have a synergistic effect, cells were treated as follows: 1) untreated, 2) PEG-SMRwt-CLU, 3) paclitaxel, 4) Paclitaxel combined with PEG-SMRwt-CLU, 5) cisplatin, 6) Cisplatin combined with PEG-SMRwt-CLU.

### Assessment of apoptosis

Breast cancer cells were seeded into 6-well plates at 4 × 10^5^ cells per well and treated with either paclitaxel or cisplatin or various concentrations of SMR peptides for 24 hours or different time point. SMR as described above. Apoptosis was determined the using AnnexinV-FITC detectction kit (Nexcelom, MA) and visualized by Cellometer imaging cytometry.

### Exosome isolation and purification

Exosomes were isolated from breast cancer cells by differential centrifugation as previously described [22]. As a control, we used untreated tumor cells. Briefly, above treated and untreated cell supernatants were centrifuged at 400 × g for 10 minutes. The supernatants were transferred to a clear tube and centrifuged at 10,000 × g for 30 minutes. The supernatants from the second spin were ultracentrifuged at 200,000 × g for 2 hours to pellet exosomes. Finally, the exosome pellets were re-suspended with PBS and stored at 4^°^C until used for analysis.

### Exosomes characterization by acetylcholinesterase (AchE) assay

Purified exosomes were quantitated by measurement of AchE as described [49]. Briefly, we prepared 100 mM dithibionitrobenzoic (DTNB) as a stock color indicator, and prepared 28.9 mg/mL in PBS of acetylthiocholine iodide as a stock substrate. Substrate stock can be stored at −20°C up to one month and color indicator can be stored at 4^°^C for two weeks. A working solution was prepared by mixing 10 mL of PBS with 200 μL of Substrate and 500 μl of DTNB. 50 μL of each exosome sample was transferred to 96 well microtitre plates, and then a standard curve prepared using AchE from 0.98 mU/mL to 2000 mU/mL. After 50 μL of standards were added into separate wells 200 μL of the working solution was added to all wells. After 20 min incubation, AchE activity was measured at 450 nm using a SpectroMax M5 fluorimeter.

### Exosome nanoparticles tracking analysis (NTA)

Analysis of absolute size distribution of exosomes was performed using NanoSight LM10 with NTA2.3 (NanoSight Ltd., Minton Park, UK). Particles were automatically tracked and sized based on Brownian motion and the diffusion coefficient. After isolation, the untreated and treated breast cancer exosomes were re-suspended in 0.5mL of PBS. Control medium and filtered PBS were used as controls in this technique. The NTA measurement conditions were: temperature = 21.0 +/− 0.5°C; viscosity = 0.99 +/− 0.01 cP, frames per second = 25, measurement time = 30s. The detection threshold was similar in all samples. Two recordings were performed for each sample.

### Western blotting

Exosomes were isolated from culture supernatants as decribed above. Protein concentration was determined by measuring absorbance at 280 nm (Nanodrop 2000) Protein samples were denatured in SDS-PAGE sample buffer by heating at 95^°^C for 15 min. Criterion TGX Precast Gels (4–20 % Bio-Rad, Richmond, CA) were used to separate the proteins and blotted as previously described [12]. Blots were incubated with the primary antibodies, anti-CD63 and anti-Alix, followed by goat or rabbit anti-Ig secondary antibodies, and Specific bands were detected using ECL chemiluminescent substrate (Santa Cruz Biotechnology, Santa Cruz, CA) and visualized on the ImageQuant LAS 4000 imaging system (GE Healthcare, Piscataway, NJ 08854).

### Fluorescent N-Rh-PE measurement

The fluorescent phospholipid analog N-Rh-PE [N-(lissamine rhodamine B sulfonyl) phosphatidyl ethanolamine] is a lipid marker of exosomes and intraluminal vesicles of multivesicular bodies as previously described [50]. Briefly, 10 mM of the N-Rh-PE was stored in chloroform/methanol (2:1). The 5 μM of N-Rh-PE with pre-cool medium mixture was then added to the treated with MCF-7 breast cancer cells transfected with siRNA-Negative or siRNA-HSPA9, and then were incubated at 4°C for 1 h. After this incubation period, the medium was removed and the cells were extensively washed with cold medium to remove excess unbound lipids. Labeled cells were cultured in complete RPMI-1640 with 10% exosome-depleted FBS medium heat inactivated at 37^°^C for overnight. To collect supernatants/exosomes and measure N-Rh-PE, we used Spectrometer at 550 nm and 590 nm excitation and emission wavelengths, respectively.

### Transfection with mortalin antibody

MCF-7 breast cancer cells were transfected with Mortlain antibody by using Chariot kit (Active Motif, Carlsbad, CA), according to the manufacturers protocol. Cells were incubated for 48 hours, after incubate period, the exosomes were isolated and measured by AchE assay and NanoSight analysis.

### Transient transfection with small interfering RNA (siRNA)

MCF-7 breast cancer cells were transfected with double-stranded siRNAs using Amaxa's Nucleofector kit (Lonza Walkersville Inc., Walkersville, MD), according to the manufacturer's protocol. Transfection of plasmids was done with Amaxa Biosystems Nucleofector II as recommended. The small interfering RNA for HSPA9 was identical to that previously decribed [25]. Following transfection, the cells were incubated at 37^°^C for 24, 48, 72 and 96 hours. Exosomes were isolated at each time point and measured by AchE assay and Western blotting.

### Statistical analysis

Data are expressed as the mean ± standard deviation (S.D.). A two-sample *t-test* assuming equal variances was used to compare the differences between controls and treated samples in each group. A value of *p* ≤ 0.05 was considered to be statistically significant.
